# Factors associated with HIV-positive status awareness among adults with long term HIV infection in four countries in the East and Southern Africa region: A multilevel approach

**DOI:** 10.1371/journal.pgph.0002692

**Published:** 2023-12-05

**Authors:** Bongi Siyabonga Nkambule, Grace Sambo, Halide Z. Aydin, Nadire Gülçin Yildiz, Kemal Aydin, Hatice Yildiz, Ichtiarini Nurullita Santri, Yuniar Wardani, Khoiriyah Isni, Bwanalori Mwamlima, Yohane Vincent Abero Phiri

**Affiliations:** 1 International Health Program, Institute of Public Health, College of Medicine, National Yang Ming Chiao Tung University, Taipei, Taiwan; 2 Chang Gung Medical Education Research Centre (CG-MERC), Chang Gung Memorial Hospital, Taoyuan, Taiwan; 3 Arnold School of Public Health, University of South Carolina, Columbia, South Carolina, United States of America; 4 Faculty of Education, Department of Guidance and Counseling, Istanbul Medipol University, Istanbul, Turkey; 5 Faculty of Economics and Administrative Sciences, Amasya University, Amasya, Turkey; 6 Health Sciences Institute, Istanbul Medipol University, Istanbul, Turkey; 7 Faculty of Public Health, Universitas Ahmad Dahlan, Yogyakarta, Indonesia; 8 Directorate of Health and Social Services, Rumphi District Council, Rumphi, Malawi; 9 Department of Epidemiology and Environmental Health (EEH), University at Buffalo, Buffalo, New York, United States of America; 10 Charis Professional and Academic Research Consultants (CPARC), Mchinji, Malawi; 11 Malawi Environmental Health Association (MEHA), Lilongwe, Malawi; National University of Singapore, SINGAPORE

## Abstract

Antiretroviral treatment (ART) appropriately and regularly used decreases the human immunodeficiency virus (HIV) viral load in the bloodstream, preventing HIV-infected people from spreading the infection to others. Disparities in ART adoption persists in East and Southern Africa, with low HIV-positive status knowledge being the primary factor. We investigated individual and household characteristics of HIV-positive status awareness among adults with long-term HIV infection in four East and Southern African countries: Eswatini, Malawi, Tanzania, and Zimbabwe. The study analyzed data from surveys conducted in Eswatini, Malawi, Tanzania, and Zimbabwe in 2015–2016. Only individuals who tested positive for HIV through rapid tests were included in the analysis. Those who already knew they were HIV-positive were categorized as aware, while those who reported being negative, never tested, or didn’t know their status were categorized as unaware. Statistical models were used to examine various factors related to HIV awareness. Pooled and country-specific odds ratios were computed. The percentage of people who knew they had HIV ranged from 58% (Tanzania and Malawi) to 87% (Eswatini). After adjusting for other variables, young persons in all countries were less likely to be aware of their HIV-positive status. Gender, marital status, education, working status, household wealth, and urbanization level of households were also associated with HIV-positive status awareness but inconsistent across countries. HIV-positive status awareness in these four East and Southern African nations remained unsatisfactory as compared to the United Nations’ 95% guideline, indicating that testing and knowledge of HIV testing in this region still has a lot of potential for improvement. The observed variations among nations may be attributable to differences in HIV pandemic culture and policies. The findings of this study will assist governments determining which subpopulations to target to boost adoption of HIV testing services, as well as in designing and development of policies.

## Introduction

In the East and Southern Africa region, the prevalence of Human Immunodeficiency Virus (HIV) remains a significant public health concern [1]. Despite extensive efforts to combat the epidemic, HIV-positive awareness rates are still very low in some countries in the region ranging between 15–98% [2]. Such poor HIV-positive awareness rates hinder efforts dedicated toward HIV prevention. HIV-positive status awareness leads to enrollment in Antiretroviral treatment (ART) [3]. When used appropriately and regularly, ART reduces an infected individual’s viral load to a suppressed level, lowering the odds of the virus spreading to the next person [4, 5]. Antiretroviral treatment (ART) as an effective HIV infection prevention has reignited optimism for HIV eradication [4]. Thus, using ART as a major HIV prevention strategy, the Joint United Nations Programme on HIV/AIDS (UNAIDS) hopes to eliminate HIV by 2030 [6]. In addition, UNAIDS has set ambitious goals for 2025, which include ensuring that 95% of individuals living with HIV are aware of their HIV-positive status, 95% of diagnosed HIV cases receive continuous antiretroviral therapy, and 95% of those on therapy achieve viral suppression. However, achieving its goal would be challenging for UNAIDS due to the persistent low awareness rates of HIV-positive status in East and Southern Africa, which happens to have the highest prevalence of HIV globally [2].

To comprehensively examine the factors influencing HIV-positive status awareness, it is essential to adopt a theoretical framework that considers the complex interplay between individual, interpersonal, community, and societal factors. The social-ecological model provides a suitable theoretical lens for understanding the multi-level influences on HIV [7]. At the individual level, factors such as knowledge about HIV/AIDS, attitudes toward testing, and perceived stigma play a crucial role in determining one’s awareness of their HIV status [8, 9]. Additionally, individual characteristics such as age, gender, marital status, education, and socioeconomic status may also influence awareness levels [10–14]. Moving beyond the individual level factors, interpersonal factors within households can significantly impact HIV-positive status awareness. Factors such as social support, household wealth, communication patterns can either hinder or facilitate individuals’ willingness to seek HIV testing and subsequently become aware of their status [15–17]. Furthermore, community level factors, including community literacy levels, residential urbanization level, community norms surrounding HIV/AIDS, and availability of testing facilities can shape individual’s awareness of their HIV-positive status [18–20]. Societal factors, such as government policies, social inequalities, cultural beliefs, also contribute to the overall context within which HIV-positive status awareness is situated [21–23]. By including the aforementioned elements in policies and strategies for promoting health behavior change and raising awareness about HIV-positive status, an environment that is supportive and inclusive is established. This environment helps to reduce stigma and motivates individuals to actively prioritize their health and overall well-being.

A few studies have combined data from multiple countries to learn more about factors that are associated with access to HIV services and care. One study examined data from three countries: Cote d’Ivoire, South Africa, and Malawi. It investigated individual level factors associated with a higher mortality rate in patients who had HIV and were already on ART [24]. Another study by Haas et al., (2020) combined data from Zambia, Eswatini, Zimbabwe, Lesotho, and Malawi in East and Southern Africa, examining the individual-level factors associated with viral load suppression in ART patients [25]. Although none of the previous multi-country research studies investigated the multi-level factors associated with access to HIV testing and care, the available data demonstrates that conducting such studies to explore access to HIV services can contribute to understanding the HIV situation in a specific region and offer learning opportunities between countries [26]. Additionally, through the utilization of a multi-level method, research studies can illuminate the intricate interaction of various factors that impact levels of awareness. This, in turn, provides valuable insights for evidence-based interventions and policies aimed at improving rates of testing and diagnosis. Although multiple HIV testing models have been implemented in the sub-Saharan region to enhance access to HIV testing, the uptake remains low [27]. Furthermore, the uneven distribution of success in various HIV testing strategies can be attributed to limitations in comprehending which strategies are most suitable for specific regions or populations [28]. Therefore, by adopting a multi-level approach this study aimed at investigating individual and household level factors associated with HIV-positive status awareness among people with long-term HIV infection in four East and Southern African countries; Eswatini, Tanzania, Malawi, and Zimbabwe.

## Methodology

### Study design, setting and data collection

Population-based HIV impact assessment (PHIA) surveys were conducted in several Sub-Saharan African countries and used as our primary data source. The PHIA household surveys are funded by the US President’s Emergency Plan for AIDS Relief (PEPFAR). These surveys are nationally representative and cross-sectional and aim to assess the state of the HIV epidemic in Sub-Saharan African countries. The PHIA surveys employed a two-stage-clustered sampling method. Firstly, enumeration areas using a probability-proportional-to-size sampling method was conducted, and then, in each area, households were randomly selected. Trained staff members interviewed household members aged 15 and above, as well as visitors who had slept in the selected households the night before. After the subjects supplied written informed consent, the interviews were conducted with computer assistance. This study was done without any incentives being given to the participants. The four countries (Eswatini, Tanzania, Malawi, Zimbabwe) that were chosen had complete data from their most recent PHIA surveys in 2015–2016. The surveys were conducted with the permission of the ethics committees in all four countries and with the approval of the institutional review board (IRB) at the Columbia University Medical Center.

### Country profiles

The four countries selected were Eswatini, Malawi, Tanzania, Zimbabwe located in the East and Southern Africa region. Eswatini is one of the smallest landlocked with a surface area of about 17 364 in Southern Africa [29]. The population of the country is about 1.1 million. Eswatini is categorized under low-middle-income countries with a gross domestic product (GDP) per capita of about $ 3424.3 [29]. Eswatini is one country with the highest prevalence (27% in those aged 15–49) of HIV globally [30]. Eswatini provides primary health care that is mostly free, but lack and shortage of resources, medicine and personnel pose accessibility barriers [31]. The total HIV expenditure is 2017 in Eswatini was $ 96 931 522 [30]. With male circumcision being another HIV prevention strategy, the proportion of males that are circumcised in Eswatini is 8.2 percent [32].

Malawi is another land lock country that shares borders with Mozambique, Zambia and Tanzania with a surface area of 118 484 square kilometers [29]. This country is categorized as a low-income country with the lowest gross domestic product (GDP) per capita among the four countries of about $ 636.8, and has a population of about 18 600 00 people [29]. Malawi has a lower prevalence of HIV, about 8.9% in people aged 15 to 49 [30]. Healthcare services in Malawi are primarily funded by the government and provided free of charge to all Malawians. Additionally, there are private facilities that operate for profit or not-for-profit, working in conjunction with the ministry of health [33]. Free ART and HIV services were rolled out in 2004 in Malawi [34]. The total HIV spending in 2017 in Malawi was $ 218 441 548. The proportion of males circumcised in Malawi is estimated to be 21.6 percent [32].

Tanzania is the biggest country among the four countries with a surface area of 947303 square kilometers [29]. Tanzania has a population of about 58 000 000 and is categorized as a low-middle-income country with GDP per capita of $ 1076.5 [29]. Tanzania has the lowest prevalence of HIV among these countries, with a prevalence of 4.8% among those aged 15 to 49 [30]. The healthcare system in Tanzania is mainly public facilities that often provide inadequate access to health care for the population due to limited resources [35]. Tanzania made ART and HIV services free for all citizens since 2004 [36]. There are about 72 percent of males estimated to be circumcised in Tanzania [32].

Zimbabwe is the second biggest country among the four countries with a surface area of 390 757 square kilometers [29]. Zimbabwe is categorized as a lower-middle-income country with a GDP of about $ 1214.5 population of about 14 645 468 people [29]. The HIV prevalence in people 15 to 49 years is 12.8% in Zimbabwe [30]. Health care services are almost free in Zimbabwe. However, due to limited resources, it is inadequate for its population [37]. HIV services and ART were also free of charge in Zimbabwe since 2003 [38]. HIV expenditure in Zimbabwe was around $ 253 479 644 in 2017 [30]. Zimbabwe is estimated to have around 9.2 percent of circumcised males [32].

### Study sample

We only included people who tested HIV positive through a rapid test in the survey and with long term HIV infection. We distinguished participants with long-term HIV infection and those with recent HIV infection based on their anti-HIV antibody avidity index values. Long-term HIV infection was defined as infection approximately more than six months. Participants who had missing values for one or more of the variables investigated in this study were also excluded from the final analysis (1 per cent). The total study sample included 10 330 people (2944 in Eswatini, 2183 in Malawi, 1742 in Tanzania, and 3461 in Zimbabwe).

### Study variables

#### Outcome measure

The key dependent variable was individuals’ knowledge of their HIV-positive status. Two questions were used to determine HIV-positive status awareness: "Have you ever been tested for HIV?" and "What was the outcome of the test?" People who reported to be HIV positive before taking the HIV rapid test during the survey were recoded as aware of their status. Those who reported being negative, or never tested before, or did not know their status were recoded as unaware of their status.

#### Independent variables

Individual factors considered in this study included age (15–25, 26–35, 36–45, 46–55 and 56+), gender (men and women), and marital status (never married and ever married). And socioeconomic status characteristics such as educational attainment (highest educational attainment, less than high school, high school level and above), and working status (yes or no if you have done any work in the last 12 months for which you have received a salary, cash, or goods as payment) were also included. Household characteristics covered the gender of the household head (man or woman), household wealth (in quintiles of Q1-Q5 ranging from low to high), and the urbanization level of the household’s residential area (urban or rural).

### Statistical analysis

HIV-positive status awareness levels according to each country were described using frequencies, and chi-square was used to assess differences between countries. We used a two-level multilevel analysis to identify characteristics associated with HIV-positive status awareness. A mixed-effects regression model with a random intercept at household level was computed. The model used pooled data from the countries to calculate adjusted odds ratios (aORs). We also did country-specific analyses due to the differences in HIV pandemic culture and practices between nations. Household weights were included in all regression models for variance estimation to account for the complex survey sampling methodology. The statistical significance was set at p<0.05. All the analyses were conducted using Stata (version 15.1; Stata Corp, College Station, TX, USA).

### Ethics statement

The PHIA surveys were conducted with the approval of ethical committees in all four nations and Columbia University Medical Center’s Institutional Review Board (IRB). Written consent was obtained from each recruited participant, or from their parent/guardian if they were under 18 years old. Participants were informed that their submitted information would be kept confidential and anonymous.

## Results

The proportion of HIV-positive people who were aware of their HIV status ranged from 58% in Tanzania to 87% in Eswatini (Table 1). The univariate analysis (Table 2) demonstrated a strong association between age and HIV-positive status awareness. In Eswatini, Malawi, and Zimbabwe, the older the age, the more likely they were to be aware of their own positive HIV status. In Tanzania, however, those over the age of 55 and young adults aged 35 and under were no more likely than the reference group to be aware of their positive HIV status (people aged at 25 years or below). All other individual and household-level characteristics were found to be significantly associated to HIV-positive status awareness.

**Table 1:** Country-specific proportions of adults knowing their own HIV positive status among those who tested positive of HIV.

Variables	Eswatini n = 2944 (%)	Malawi n = 2183 (%)	Tanzania n = 1742 (%)	Zimbabwe n = 3461 (%)	*p-value* ^c^
**HIV-positive status awareness**					<0.001
**Yes**	2558 (87)	1269 (58)	1016 (58)	2198 (64)	
**No**	386 (13)	914 (42)	776 (42)	1263 (36)	

^c^ Chi-Square test p-values, Test for significance set at p<0.05

**Table 2:** Univariate analyses for HIV positive awareness among adults with long term HIV infection.

Variables	Pooled uOR(95%CI)	Eswatini uOR(95%CI)	Malawi uOR(95%CI)	Tanzania uOR(95%CI)	Zimbabwe uOR(95%CI)
**Age (15–25)**					
26–35	1.48** (1.10–1.99)	2.73*** (1.82–4.09)	2.14*** (1.47–3.11)	0.98 (0.44–2.15)	1.52** (1.12–2.07)
36–45	3.79*** (2.71–5.30)	3.97*** (2.53–6.22)	5.26*** (3.59–7.69)	2.39* (1.05–5.45)	5.18*** (3.65–7.34)
46–55	6.94*** (4.52–10.65)	6.44*** (3.67–11.29)	9.19*** (5.87–14.38)	4.00** (1.46–10.95)	12.47*** (8.08–19.25)
56+	4.13*** (2.70–6.35)	5.51*** (3.08–9.87)	12.28*** (6.87–21.94)	1.11 (0.43–2.84)	10.32*** (6.53–16.29)
**Gender (Female)**					
Males	1.20* (1.01–1.42)	0.38*** (0.28–0.52)	2.02*** (1.57–2.60)	0.45** (0.28–0.72)	2.03*** (1.65–2.49)
**Marital status (Never married)**					
Ever married	1.14 (0.86–1.50)	2.73*** (2.03–3.66)	1.74* (1.14–2.65)	1.01 (0.45–2.26)	1.43* (1.08–1.90)
**Educational level (<High school)**					
≥High school	0.99 (0.71–1.40)	0.62** (0.46–0.82)	1.09 (0.65–1.82)	0.71 (0.34–1.48)	0.94 (0.59–1.50)
**Working status (Not working)**					
Working	0.79** (0.67–0.93)	0.78 (0.61–1.00)	0.99 (0.80–1.23)	0.49** (0.30–0.78)	0.94 (0.79–1.11)
**Household head (Female)**					
Male	0.78* (0.65–0.94)	0.71* (0.53–0.94)	1.06 (0.86–1.30)	0.55* (0.32–0.93)	1.02 (0.86–1.21)
**Household wealth (Q1)**					
Q2-Q4	1.36** (1.09–1.71)	0.62** (0.44–0.88)	1.39* (1.06–1.81)	2.64** (1.33–5.24)	0.87 (0.70–1.08)
Q5	1.49** (1.12–1.96)	0.48** (0.29–0.78)	1.31 (0.95–1.81)	3.33** (1.45–7.65)	1.00 (0.78–1.29)
**Residence (Rural)**					
Urban	1.14 (0.94–1.37)	0.51*** (0.38–0.69)	0.92 (0.75–1.13)	1.72* (1.04–2.82)	1.09 (0.90–1.32)

*p < 0.05;

**p < 0.01;

***p < 0.001;

uOR: Unadjusted Odds Ratio

In the multivariate analysis (Table 3), we observed that age was positively associated with HIV-positive status awareness. Individuals of older age were more likely to be aware of their HIV-positive status. Moreover, gender differences in HIV-positive status awareness were observed across all countries studied, albeit with varying directions. In Eswatini and Tanzania, men were significantly less likely to be aware of their HIV-positive status than women, aOR 0.29 (95% CI 0.20–0.42) and aOR 0.50 (95% CI 0.29–0.86), respectively. However, in Malawi and Zimbabwe, men were significantly more likely to be aware of their HIV-positive status, aOR 1.73 (95% CI 1.30–2.29) and aOR 1.68 (95% CI 1.33–2.12), respectively.

**Table 3:** Multivariate analysis of factors associated with HIV-positive status awareness among adults with long term HIV infection.

Variables	Pooled aOR (95%CI)	Eswatini aOR (95%CI)	Malawi aOR (95%CI)	Tanzania aOR (95%CI)	Zimbabwe aOR (95%CI)
**Age (15–25)**					
26–35	1.82*** (1.33–2.50)	2.81*** (1.80–4.40)	2.26*** (1.47–3.48)	1.15 (0.47–2.77)	2.20*** (1.53–3.16)
36–45	4.67*** (3.26–6.70)	4.58*** (2.80–7.50)	5.37*** (3.31–8.76)	3.13* (1.17–8.36)	7.45*** (4.88–11.40)
46–55	8.38*** (5.37–13.06)	6.80*** (3.66–12.61)	8.83*** (5.00–15.58)	5.28** (1.61–17.31)	17.52*** (10.61–28.91)
56+	4.93*** (3.15–7.73)	5.10*** (2.60–9.99)	12.09*** (6.00–24.39)	1.30 (0.43–3.92)	14.08*** (8.33–23.79)
**Gender (Female)**					
Males	1.09 (0.90–1.32)	0.29*** (0.20–0.42)	1.73*** (1.30–2.29)	0.50* (0.29–0.86)	1.68*** (1.33–2.12)
**Marital status (Never married)**					
Ever married	0.64** (0.47–0.87)	1.86*** (1.35–2.57)	0.88 (0.55–1.40)	0.76 (0.30–1.92)	0.52** (0.35–0.77)
**Educational level (<High school)**					
≥High school	1.22 (0.85–1.75)	0.70* (0.50–0.97)	0.97 (0.53–1.75)	0.88 (0.38–2.05)	0.93 (0.55–1.57)
**Working status (Not working)**					
Working	0.72** (0.60–0.87)	0.89 (0.66–1.20)	0.85 (0.66–1.09)	0.50** (0.31–0.83)	0.88 (0.72–1.08)
**Household head (Female)**					
Male	0.82* (0.67–0.99)	0.96 (0.69–1.32)	0.89 (0.70–1.14)	0.80 (0.45–0.83)	0.85 (0.68–1.06)
**Household wealth (Q1)**					
Q2-Q4	1.32* (1.03–1.68)	0.78 (0.52–1.15)	1.25 (0.93–1.69)	2.49* (1.17–5.30)	0.82 (0.64–1.05)
Q5	1.32 (0.92–1.89)	0.79 (0.44–1.41)	1.17 (0.75–1.83)	2.50 (0.90–6.93)	0.85 (0.57–1.26)
**Residence (Rural)**					
Urban	1.09 (0.85–1.40)	0.57** (0.40–0.80)	1.02 (0.77–1.36)	1.35 (0.73–2.51)	1.28 (0.93–1.76)
**Variance for household**	**1.93**	**1.72**	**0.07**	**7.49**	**1.05**
**ICC for household**	**0.37**	**0.32**	**0.02**	**0.69**	**0.24**

*p < 0.05;

**p < 0.01;

***p < 0.001;

aOR: Adjusted Odds Ratios; ICC; Intra-class correlation

The other significant factors associated with HIV-positive status awareness were marital status, education, and working status. However, this was not always the case in all countries. In Eswatini, people who ever got married were more likely (aOR = 1.86; 95% CI [1.35–2.57]) to be aware of their HIV-positive status than those who never got married. However, married individuals were significantly less likely to be aware of their status than those who never married in Zimbabwe (aOR = 0.52; 95% CI [0.35–0.77]). In terms of socioeconomic status, those with higher education levels were considerably less likely to be aware of their HIV-positive status than those with lower education levels in Eswatini (aOR = 0.70; 95% CI [0.50–0.97]). Working people were less likely than those who did not work to be aware of their HIV-positive status, but the difference was only statistically significant in Tanzania (aOR = 0.50; 95% CI [0.31–0.83]).

Individuals from homes headed by men consistently showed to be less likely to be aware of their HIV-positive status, although the difference approached statistical significance only in the pooled analyses. The association between family wealth and HIV-positive status awareness varied by country. Only in Tanzania where individuals from affluent families (Q2-Q4) were more likely to be aware of their HIV-positive status than people from impoverished households (Q1) (aOR = 2.49; 95% CI [1.17–5.30]). Also, Eswatini was the only country where there was a difference in HIV-positive status awareness between urban and rural areas. People in Eswatini’s urban regions were much less likely to be aware of their HIV-positive status than those in rural areas (aOR = 0.57; 95% CI [0.40–0.80]). The random intercept multilevel model indicated that the interclass correlation coefficient (ICC) in the pooled null model was 0.37, suggesting that approximately 37% of the total variation in ART enrollment was attributable to observed and unobserved characteristics at the household level.

## Discussion

The Sub-Saharan region has witnessed substantial improvements and progress in terms of HIV/AIDS awareness levels throughout the years, which may be attributed to extensive public health campaigns and educational initiatives, among other factors [39]. Nevertheless, there are still disparities in awareness levels that continue to exist within the region. In this study, awareness of HIV-positive status ranged from 58% (Tanzania, Malawi) to 87% (Eswatini), suggesting that testing remain a serious challenge to HIV care in this region. Some studies suggest that the low awareness may be an indication of low prevalence of HIV testing in the East and Southern Africa region [40]. Young people were consistently found to be at risk of not knowing their own positive HIV status in all countries. Other factors like gender, marital status, education, working status, household wealth, and household settlement were associated with HIV-positive status awareness but the direction of the associations was not consistent across countries. In this study, we found that young people were less likely than older persons to be aware of their HIV-positive status. Consistent with other research [10, 41], the reason behind the lack of awareness of HIV-positive status among young individuals could be attributed to their overall limited utilization of healthcare services. Furthermore, HIV-related stigma may hamper their motivation to seek HIV testing services, fearing being labeled as having been exposed to promiscuous sexual behaviors [41].

We also demonstrated that men were significantly less likely to be aware of their HIV-positive status in Eswatini and Tanzania which is consistent with results from other previous studies [14, 40]. Men are reported to use health care services less than women, which may result in less interactions with health care systems and, as a result, less exposure or chance to HIV testing services [42]. Furthermore, programs such as prenatal care, which requires women to get tested for HIV on a regular basis, boost the testing rate for women [43]. Additionally, attributable to gender and cultural norms, men are less likely to get tested for HIV, because they share the belief that their partner’s HIV results are their own [44]. Working and being busy at work might also explain why males were less aware of their HIV status in our study. Men may be less able to attend testing services owing to more rigorous employment requirements, and the opening hours of these testing services may not meet their work schedule [14].

Directly opposed to the findings above, we did observe that Malawian and Zimbabwean men were more likely than women to be informed of their HIV-positive status. Increasing HIV-positive status awareness might be attributed to increased HIV testing initiatives focused on working-class groups or contact indexing in Zimbabwe and Malawi [39, 45]. Furthermore, recent evidence demonstrates that Malawi has made significant progress in achieving the UNAIDS 95:95:95 targets [39]. The findings indicate that out of the three targets, namely, 95% Awareness, 95% ART Coverage, and 95% Viral Suppression, the latter two have been achieved. Notably, there has been a steady increase in the number of men who are aware of their HIV status, and the average HIV-awareness in the country currently ranges from 70% to 91% depending on the region. Research conducted in six Sub-Saharan African nations revealed that 19–54% of men had never undergone HIV testing. Nevertheless, during the study, a remarkable 85–99% of the men willingly agreed to participate in the testing process [14]. In Botswana, a cluster randomized control study was conducted to provide HIV testing to males through mobile services and home-based testing in accessible venues. The study revealed a 37% increase in HIV status awareness among men, compared to a 19% increase among women [46]. It is possible that the rising awareness of HIV-positive status in these countries could be attributed to the expansion of HIV testing services in locations frequently visited by men.

Inconsistent findings across countries were observed in the relationship between marital status and HIV-positive status awareness. In Zimbabwe, those who had ever married were less likely to be aware of their HIV-positive status. Evidence demonstrates that in some communities where HIV stigma is higher, married persons may be afraid of divorce and loss of support if they disclose their HIV status [47–49]. In Eswatini, however, married persons were more likely than unmarried people to be aware of their HIV-positive status. Because of partner participation and support, married persons may be more aware of their HIV status [50]. Since 2009, Eswatini has been running the Love Test campaign under the Population Services International organization [51]. By portraying HIV testing as an act of love, the Love Test campaign attempted to increase HIV testing among couples, with a specific target of increasing male involvement in various HIV services.

Working persons in Tanzania were less likely to be aware of their HIV status than those who did not work. This might imply that being busy at work influences not just ART enrolment but also HIV testing among working persons [52]. People in Eswatini with less education and from rural regions were more aware of their HIV-positive status than those with higher education and from urban areas. Over the last few years, intensive HIV campaigns and a greater focus on battling HIV among the socially excluded and those from rural regions may have contributed to the increasing HIV-positive awareness among these subpopulations [53]. In Tanzania, individuals from poor households had lower awareness than those from rich households. One probable cause might be unequal access to testing facilities between persons from poor and wealthier households. In Tanzania, for example, clinics that serviced disadvantaged communities were frequently reported to have a personnel deficit and lacked equipment such as HIV test kits [54].

Our study had some strengths and limitations to take into consideration. This study is one of the few that uses nationally representative data from many countries and a multilevel analytical technique to evaluate factors associated with HIV positive self-awareness in adults with long-term HIV infection. Furthermore, our study expanded on previous research by specifically investigating awareness of one’s own HIV-positive status among people with long HIV infection. Previous studies have investigated factors associated with HIV-positive awareness but did not distinguish between those with long and recent HIV infection.

Nonetheless, several limitations should be considered when interpreting our findings. First, we only included a small number of East and Southern African nations since data in other East and Southern African countries were not accessible for public use when this study began. Also, due to data availability and for comparison purposes, we used 2015–2016 data. We acknowledge that some countries in our sample may now have higher HIV-positive status awareness rates. Recent updates on the Population-Based HIV Impact Assessment (PHIA) reveal that out of the four countries included in our study, only Zimbabwe has up-to-date data available for public access (https://phia.icap.columbia.edu/). Although studies have been completed for Malawi and Eswatini in 2020–2021 and 2021 respectively, the data has not yet been made publicly accessible. In Tanzania, the most recent available data is from 2016–2017. Therefore, we consider the presented results to be significant as they allow for comparisons between different time periods and regions and enable evaluation of the effectiveness of past policies and interventions in raising awareness of HIV-positive status. Additionally, the pooled study results may not always provide valuable insights due to conflicting relationships between certain characteristics and HIV-positive status awareness. These differences could be attributed to cultural variations and disparities in HIV prevention practices across different countries. Moreover, our study being cross-sectional made it challenging to establish temporal associations between the dependent variable and various independent variables. Furthermore, the self-reported nature of our study’s outcome (HIV-positive awareness) introduces the possibility of social desirability bias. There is a likelihood that individuals who were aware of their HIV-positive status might have claimed to be unaware, particularly if they were not already receiving ART.

## Conclusion

We demonstrated that the level of awareness regarding HIV-positive status differs among countries in the sub-Saharan region, and that it falls below the recommended guidelines set by UNAIDS. This suggests that tailored HIV testing programs may be more effective in specific areas compared to others in the region studied. Factors such as age, gender, and employment status were identified as potential reasons for lower HIV awareness. Additionally, limited access to HIV care among certain groups might be attributed to inadequate healthcare-seeking behavior. Home-based and community-based testing and ART initiation could be effective approaches to improve access to HIV care among people with poor healthcare-seeking behavior (young people, males, and working people) in East and Southern Africa [46, 55, 56]. However, the study also found that socioeconomic differences in HIV-positive status awareness reduced in the East and Southern regions. This suggests that governments and non-governmental organizations in these areas should persist in their efforts to enhance the accessibility and affordability of HIV testing for marginalized individuals.
